# Assessment of the relationship between metabolic syndrome and obstructive sleep apnea in male drivers of Shahroud city in 2018: a cross sectional study

**DOI:** 10.1186/s12889-019-7361-5

**Published:** 2019-08-06

**Authors:** Mina Shayestefar, Khosro Sadeghniiat Haghighi, Shayesteh Jahanfar, Mehri Delvarianzadeh, Farzaneh Nematzadeh, Mohammad Hossein Ebrahimi

**Affiliations:** 1 School of Allied Medical Sciences, Semnan University of Medical Sciences, Semnan, Iran; 2 Occupational Sleep Research Center, Tehran University of Medical Sciences, Tehran, Iran; 3 MPH Program, School of Health Science, Central Michigan University, Mount Pleasant, USA; 4 School of Public Health, Shahroud University of Medical Sciences, Shahroud, Iran; 5 Student Research Committee, School of Medicine, Shahroud University of Medical Sciences, Shahroud, Iran; 6 Environmental and Occupational Health Research centre, Shahroud University of Medical Sciences, Shahroud, Iran

**Keywords:** Professional drivers, Metabolic syndrome, Sleep disorders

## Abstract

**Background:**

Metabolic syndrome involves a set of metabolic risk factors that directly increases the risk of atherosclerotic cardiovascular disease. Physical inactivity due to driving can increase the risk of metabolic syndrome. It is also known that sleep disorders (sleep apnea) can result in MetS. Driving in Iran is considered a very popular but risky occupation, so paying attention to this profession is of special importance. Therefore, the researchers aimed to investigate the association between sleep disorders and metabolic syndrome in drivers in Shahroud city in 2018.

**Methods:**

This cross-sectional study was carried out on 948 drivers from Shahroud city in 2018. After obtaining consent from participants, 3 questionnaires including demographic, Epworth Sleepiness Scale and STOP-BANG were completed. Clinical and anthropometric measurements were assessed, including blood pressure, waist circumference, hip circumference, weight, height, and body mass index. In addition, blood was drawn to measure High Density Lipoprotein, Low Density Lipoprotein, Triglyceride, cholesterol, and Fasting Blood Sugar levels. The relationship between metabolic syndrome and sleep disorders was then studied. In this study, statistical analyses were performed using SPSS software version 23 at a significance level of 0.05.

**Results:**

Mean age of drivers was 44.15 ± 11.66 (years). The mean waist circumference and mean hip circumference in subjects with a Class 1 Driver’s License (a certificate for trucks and buses) were higher than those with a Class 2 Driver’s License (a certificate for motorcars, minibuses, vans, etc. (seating< 20)) (*P* = 0.01 and *P* = 0.003, respectively). Moreover, the BMI in subjects with a Class 1 Driver’s License was higher compared to subjects with a Class 2 Driver’s License. The correlation between metabolic syndrome with sleep apnea based on STOP-BANG questionnaire was significant (*p* < 0.001) irrespective of definition (ATP and IDF).

**Conclusions:**

According to the results of this study, there was a bi-directional association between sleep disorders and Mets, so this group should pass periodic medical examinations and training courses. Moreover, their families should be informed of prevention and treatment of this syndrome.

## Background

Metabolic syndrome (MetS) has attracted global attention in recent years [1, 2]. MetS involves a group of metabolic risk factors that directly increases the likelihood of developing atherosclerotic cardiovascular disease (ASCVD) [3].

According to the WHO, by 2020, chronic non-communicable diseases will account for three-quarters of deaths in developing countries. Changes in dietary patterns, decreased physical activity, and increased smoking are the main causes of non-communicable diseases in recent years [4].

MetS is the one of the most important health problems in the twenty-first century, and shows a growing trend in developed and developing countries. According to ATP III, cardiovascular disease is the first outcome of MetS [5]. Cardiovascular diseases are the leading cause of disability and early deaths worldwide (more than 17.1 million deaths annually since 2010), as well as huge healthcare costs [6]. The causes of the syndrome are unknown; most patients are elderly, obese, with little physical activity and some degree of insulin resistance. The most important affecting factors are genetic background; obesity, especially abdominal obesity; lifestyle; little physical activity; stress and smoking [5].

Over the past 20 years, unlike in the developed countries, the rate of cardiovascular mortality has increased by 20–45% in Iran [7]. MetS is one of the major causes of this trend. In addition, MetS increases the risk of diabetes threefold, so the diagnosis of this syndrome plays a crucial role in the prevention of diabetes and cardiovascular diseases [1].

Unfortunately, the prevalence of MetS has increased in recent years. Many studies have shown that the prevalence of MetS in various countries was between 12.8 and 41.7%, while in Iran it was reported to be between 22 to 31% [8, 9]. The prevalence of MetS in the northern region of Iran was 26.1% according to the ATP III definition, 35.2% according to the IDF definition, and 31.6% according to the AHA definition [10].

MetS prevalence among adults was mostly seen in elderly persons who were overweight or obese [11]. Insulin resistance, abdominal obesity, atherogenic dyslipidemia, endothelial dysfunction, genetic predisposition, hypertension, hypercoagulable state, and chronic stress are among the factors that make up metabolic syndrome [12].

Endothelial dysfunction, sleep duration, and duration of physical inactivity are all associated with MetS [13–16]. Most researchers reported the positive effect of physical activity on MetS [17–20].

Recent studies have shown that subjective assessment of longer sleep latency, sleep apnoea syndrome, poor sleep characteristics and quality associated are highly associated with MetS scores [21–24].

Sufficient sleep is vital for physical and mental health. However, numerous adults suffer from many problems associated with inadequate sleep or poor sleep — from cognitive deficits and behavioural problems to obesity and life-threatening incidents. Therefore, recognizing and treating sleep problems are necessary to promote the adults’ health [25].

Road accidents in Iran are the third leading cause of disability and early death, which has become a public health concern. The mortality rate in road accidents in Iran is higher than in other countries [26]; road accidents account for 30% of all accidents in the country. According to the World Health Organization (WHO), Iran ranked first worldwide in terms of road accidents [27].

Most driving accidents are directly related to human factors [28, 29]; controlling this is one of the most important factors in reducing injuries and death [30]. Given the sensitivity of driving, improving the sleep health of drivers can have a significant effect on reducing mortality and morbidity [31], as drowsiness can increase the risk of road accidents up to six times [32, 33].

Due to their sedentary life style, drivers are more susceptible to diseases, especially to the components of MetS and its complications. Lack of physical activity, the shift work, and changes in dietary habits are among the main causes of health hazards in drivers [34].

Driving in Iran is considered as a very popular but risky occupation, so paying attention to this profession is of special importance. Therefore, the researchers decided to investigate the association between sleep disorders and MetS in drivers in Shahroud city in 2018.

## Methods

After receiving approval from Occupational Sleep Research Center of Tehran University of Medical Sciences ethics committee, this cross-sectional study was conducted on 948 referred male drivers to Kasra occupational health center for annually health check-up from Shahroud city in 2018. Eligibility criteria were male sex, having mental health and consent to study participation. Female were excluded from this study because a female driver with a Class 1 Driver’s License (a certificate for trucks and buses) and a Class 2 Driver’s License (a certificate for motorcars, minibuses, vans, etc. (seating< 20)) in Iran is rare. In fact, we could only find one women with such credentials. After obtaining the written informed consent of the participants, 3 questionnaires including demographic, Epworth Sleepiness Scale (ESS) and STOP-BANG^1^ were given to them. Clinical and anthropometric measurements were assessed including blood pressure, waist circumference, hip circumference, waist to hip ratio, weight, height, and body mass index (BMI). Laboratory tests include HDL, LDL, TG, cholesterol, and FBS^2^ were done after 12 h of fasting.

### Definition of MetS

As there are multiple definitions of metabolic syndrome from different perspectives, in this study it is considered based on the IDF and ATP definitions. It should be noted that since all subjects were male, the range of risk factors in the table below are only for men (Table 1).

**Table 1:** Definitions of Metabolic Syndrome [35]

	Modified NCEP ATP III [36–38]	IDF [39]
Absolutely required	None	Central obesity (waist circumference) ≥ 94 cm
Criteria	Any three of the five criteria Below	Obesity, plus two of the four criteria below
Obesity	Waist circumference: > 40 in. (102 cm)	Central obesity already required
Hyperglycemia	Fasting glucose≥100 mg/dl or Rx^a^	Fasting glucose≥100 mg/dl or Rx
Plasma triglycerides	TG ≥150 mg/dl or Rx	TG ≥150 mg/dl or Rx
HDL cholesterol	< 40 mg/dl or Rx	< 40 mg/dl or Rx
Hypertension	> 130 mmHg systolic or > 85 mmHg diastolic or Rx	> 130 mmHg systolic or > 85 mmHg diastolic or Rx

*NCEP: ATP III* National Cholesterol Education Program’s Adult Treatment Panel III

*IDF* International Diabetes Federation

^a^Rx, pharmacologic treatment

The Blood pressure of all subjects was measured from the right arm using a mercury sphygmomanometer after at least 5 minutes rest and was repeated at a minimum five-minute interval, then the mean pressure was used. The waist circumference was measured with a measuring tape in a horizontal plane around the abdomen at the level of the iliac crest and at the end of a normal expiration of the subject; the hip circumference was measured by locating the measuring tape on the widest part of hips without pressure and with 1 cm accuracy. The weight of subjects was measured with minimum clothing and no shoes using a digital scale with 100 g accuracy. Subjects’ height was measured with 1 cm accuracy by a measuring tape while they were in the standing position next to the wall and their shoulders were in normal position.

### Epworth sleepiness scale (ESS)

The ESS is a questionnaire with eight questions which examine the quality of sleepiness in different situations. A score from 0 to 8 indicates normal sleepiness; a score from 9 to 12 shows mild sleepiness; a score of 13 to 16 demonstrates moderate sleepiness; and a score of more than 16 indicates severe sleepiness.

The validity and reliability of the Epworth Sleepiness Scale were studied by Sadeghtniat et al. in 2012. Its reliability has been evaluated in two ways. Internal consistency has been assessed using Cronbach’s alpha coefficient, which was 0.81. The test-retest reliability (the reliability of a test measured over time) was done on the results of questionnaires answered by 123 patients twice at different times: the first test was done during the patients’ first visits to a sleep clinic, and the re-test was done before their polysomnography. The interval was between 2 to 4 weeks. The validity of the questionnaire was evaluated in three ways: construct validity, criterion validity, and discriminant validity [40].

### STOP-BANG^3^ questionnaire

This questionnaire comprises eight dichotomous (yes/no) items: Snoring, Tiredness, Observed apnea, high blood Pressure, BMI more than 30, Age older than 50, Neck circumference more than 40 cm and male Gender. The questionnaire predicts the likelihood of Obstructive Sleep Apnea (OSA) and respiratory complications after surgery [41]. Having 3 or more positive answers to the 8 questions indicates a high risk of OSA; having fewer than 3 positive responses shows a lower risk of OSA. Thus, the score ranges from 0 to 8 [42, 43].

In 2015, to assess the validity and reliability of the Farsi version of the STOP-BANG questionnaire, Sadeghniat et al. studied 603 patients admitted to the sleep clinic and subjected to polysomnography. The analysis of reliability showed that 124 patients had similar scores on whole questionnaire and 130 patients were in the same level of OSA. Based on the polysomnography of 438 patients, 20.4% of them had mild, 18.9% had moderate, and 33.3% had severe OSA. It was concluded that the Persian version of the questionnaire had the same function as the original one [42].

### Statistical analysis

Univariate analysis involving descriptive statistics, t-test, chi-square, and logistic regression were carried out using SPSS software version 23 at a significance level of 0.05.

## Results

In this study, 948 drivers with the mean age of 44.15 ± 11.66 were recruited; the minimum age was 22 and the maximum age was 69 years. Six hundred twenty-two drivers (68.8%) had a Class 1 Driver’s License (a certificate for trucks and buses); 281 drivers (29.6%) had a Class 2 Driver’s License (a certificate for motorcars, minibuses, vans, etc. (seating< 20)); and 15 drivers (1.6%) had a Class 3 Driver’s License. Subjects were divided into two groups, 229 subjects (24.9%) were drivers of passenger cars and 719 subjects (75.8%) were drivers of road vehicles. About marital status, 894 subjects (94.3%) were married, and 54 subjects (5.7%) were single. The mean number of children per driver was 2.49 ± 1.16, with a minimum of 1 and a maximum of 7. There were 192 (20.25%) smokers among drivers and the mean number of used pack-year was 7.13 ± 9.25. Also waist to hip ratio was calculated and the mean of this ratio was estimated (0.95 ± 0.06).

According to the ESS, the prevalence of sleepiness was seen at both normal and mild levels in 832 subjects (87.8%) and 116 subjects (12.2%) respectively; no moderate or severe sleepiness was seen in the drivers. Based on the findings of the STOP-BANG questionnaire, a low risk of OSA was found in 712 drivers (75.1%) and a high risk of OSA was seen in 236 drivers (24.9%). Moreover, based on the definitions of ATP and IDF, the prevalence of MetS was found to be 392 (41.4%) and 497 (52.4%), respectively.

The relationship of the demographic and clinical variables with both definitions of MetS is shown in Table 2; as the relationship of the variables with the risk of breathing cessation is summarized in Table 3.

**Table 2:** Prevalence of MetS based on ATP and IDF definitions and their relationship with demographic and clinical variables

Variable		MetS (according to IDF)		Chi-square test (χ^2^)	*P*-value	MetS (according to ATP)		Chi-square test (χ^2^)	*P*-value
		Yes(%)	No(%)			Yes	No		
Class of driver’s license	Class 1	37.97	30.80	8.94	0.05	295	357	14.12	< 0.001
	Class 2	14.03	15.61			94	187		
	Class 3	0.42	1.16			3	12		
Driving Type	Passenger cars	13.5	10.65	1.45	0.11	92	137	0.172	0.35
	Road vehicles	38.92	36.92			300	419		
Marital status	Married	50	44.30	2.22	0.06	377	517	4.39	0.01
	Single	2.43	3.27			15	39		
Age	< 30	3.48	6.22	27.972	< 0.001	26	66	36.482	< 0.001
	30–40	10.55	13.71			71	159		
	40–50	19.62	15.82			138	198		
	> 50	18.57	11.81			157	133		
BMI	< 18.5	0	2.11	216.88	< 0.001	2	18	167.372	< 0.001
	18.5–25	7.38	25.42			55	256		
	25–30	26.37	15.08			174	219		
	> 30	18.67	4.96			161	63		
HTN	Yes	32.59	12.66	120.71	< 0.001	276	153	170.71	< 0.001
	No	19.83	34.92			116	403		
HDL	< 40	45.25	36.5	14.60	< 0.001	343	432	14.80	< 0.001
	≥40	7.17	11.08			49	124		
TG	≥150	10.76	34.49	177.93	< 0.001	139	290	222.625	< 0.001
	< 150	36.81	17.93			417	102		
FBS	≥100	20.04	6.54	72.616	< 0.001	188	64	156.501	< 0.001
	< 100	32.38	41.03			204	492

**Table 3:** Prevalence of OSA and sleepiness and their relationship with demographic and clinical variables

Variable		Risk of OSA		Chi-square test (χ^2^)	*P*-value	Sleepiness		Chi-square test (χ^2^)	*P*-value
		Low(%)	High(%)			normal	mild		
Class of driver’s license	Class 1	50.32	18.46	4.23	0.06	568	84	2.53	0.14
	Class 2	0.24	6.12			249	32		
	Class 3	1.27	0.32			15	0		
Driving type	Passenger cars	18.67	5.49	0.772	0.18	194	35	2.611	0.05
	Road vehicles	56.43	19.41			638	81		
Marital status	Married	70.04	24.26	5.818	0.01	782	112	1.243	0.13
	Single	5.06	0.63			50	4		
Age	< 30	9.07	0.63	220.876	< 0.001	86	6	10.902	0.006
	30–40	22.57	1.69			212	18		
	40–50	29.96	5.49			284	52		
	> 50	13.5	17.09			250	40		
BMI	< 18.5	2	0.11	161.142	< 0.001	17	3	4.023	0.12
	18.5–25	31.22	1.58			271	40		
	25–30	30.59	10.86			339	54		
	> 30	11.29	12.34			205	19		
HTN	Yes	30.38	14.87	26.639	< 0.001	385	44	2.860	0.04
	No	44.73	10.02			447	72		
HDL	< 40	62.24	19.51	2.38	0.06	676	99	1.144	0.14
	≥40	12.87	5.38			156	17		
TG	≥150	31.54	13.71	12.260	< 0.001	380	49	0.484	0.24
	< 150	43.57	11.18			452	67		
FBS	≥100	58.33	15.08	26.48	< 0.001	609	87	0.170	0.34
	< 100	16.77	9.81			223	29		

As shown in Table 2, MetS, based on the ATP definition, had a significant relationship with the class of driver’s license and marital status. This association is probably due to BMI differences. As well, MetS, according to both the ATP and IDF definitions, had a significant relationship with age, BMI, HDL, TG, FBS, and HTN (*P* < 0.05).

As mentioned earlier, the level of sleepiness in drivers was found at both normal and mild levels, and there were no moderate and severe levels of sleepiness; normal and mild levels are shown above. In addition, as shown in Table 3, marital status, BMI, TG, and FBS had a significant relationship with OSA; driving type had a significant relationship with sleepiness; and age and HTN had a significant relationship with OSA and sleepiness (*P* < 0.05).

The correlation of quantitative clinical variables including blood pressure, HDL, FBS, TG, and quantitative variables of age and BMI with the scores of ESS questionnaire and with both definitions of MetS are shown in Table 4.

**Table 4:** Correlation of clinical and demographic variables with scores of ESS questionnaire

Variable	Mean ± SD	ESS	
		R	*P* value
Age	44.18 ± 11.57	0.069	0.017
Number of children	2.49 ± 1.16	0.019	0.29
Pack/year	7.13 ± 9.25	−0.006	0.46
BMI	27.02 ± 5.05	−0.008	0.399
HTN	130.58 ± 17.58	−0.027	0.206
FBS	94.62 ± 21.14	0.006	0.430
HDL	37.78 ± 7.74	−0.033	0.154
TG	166.93 ± 81.57	−0.016	0.310

The results of Table 4 demonstrate that there was a positive and significant correlation between age and the scores of the questionnaire.

According to the results of this study, drivers with MetS suffer from more sleep disorders. It is our believe that in fact, there is a bi-directional association between sleep disorders and Mets. The correlation between metabolic syndrome with sleep apnea based on STOP-BANG questionnaire was significant (*p* < 0.001) irrespective of definition (ATP and IDF), but there was no correlation with sleepiness level based on ESS questionnaire.

In order to assess the variables affecting MetS based on the ATP and IDF definitions, a logistic regression test in a stepwise method have been used. The results for the ATP definition are shown in Table 5, and those for the IDF definition are shown in Table 6.

**Table 5:** Factors affecting MetS (ATP definition) based on logistic regression in a stepwise procedure

Variable	SE	OR	*P* Value	OR (CI%95)	
				Lower	Upper
BMI	0.044	1.235	< 0.001	1.134	1.345
Number of children	0.143	1.442	0.011	1.089	1.909

**Table 6:** Factors affecting MetS (IDF definition) based on logistic regression in a stepwise procedure

Variable	SE	OR	*P* Value	OR (CI%95)	
				Lower	Upper
BMI	0.043	1.220	< 0.001	1.121	1.327
Number of children	0.156	1.442	0.019	1.063	1.957

## Discussion

According to results, there was a significant relationship between the amount of sleepiness and the type of driving, which could be due to the fact that most drivers with a Class 1 Driver’s License had a higher BMI than other drivers. There was also a positive and significant correlation between the number of children, BMI, laboratory tests and the scores on the STOP-BANG questionnaire.

In the present study, STOP-BANG scores were significantly correlated with the number of years participants had smoked. Also most drivers with a Class 1 Driver’s License were smokers.

In line with the findings of this study, Guglielmi et al. (2017) in a study on 526 truck drivers found that 51.1% of the subjects were at risk of OSA; a high prevalence of sleep problems was seen in this population [44].

In 2016, Anund et al. investigated the factors associated with sleepiness in urban bus drivers. Their results showed that 19% of drivers struggled to stay awake and suffered from severe sleepiness 2 to 3 times a week or more [45]. However, in the present study, sleepiness based on the STOP-BANG score had a significant relationship with the type of vehicle, but this relationship was not seen based on the ESS score.

In addition, in a study on 1413 freight train and passenger train drivers, Gu et al. (2010) found that 48.43% of them had poor sleep quality; their sleep quality was significantly related to occupation, sport, and cigarette and alcohol consumption, but it had no significant relationship with the level of education, marital status, or age [46]. However, Soleimanloo et al. (2017) declared that sleep-related crashes were most common in young drivers (18–24 years); this could be due to less time to sleep, less tolerance for sleepiness, and the ongoing development of the part of the brain involved in decision making [47].

In the current study, MetS based on ATP definition had a significant relationship with the class of driver’s license and marital status; as well, MetS based on both ATP and IDF definitions had significant relationships with age, BMI, HDL, TG, FBS and HTN. Moreover, in the logistic regression analysis, BMI, number of children, and the number of years the driver had smoked were predictive factors for MetS.

In Huang et al.’s study (2016), by holding the drivers’ age constant, it was found that smoking was a risk factor for MetS in professional drivers [48]. In addition, in a study on 12,138 Iranian professional drivers of long vehicles, Mohebbi et al. (2012) using logistic regression suggested that there was a significant relationship between MetS and BMI, smoking, age, driving time per week, and driving experiences [49].

In this study, the correlation between both definitions of metabolic syndrome (ATP and IDF) with sleep apnea based on STOP-BANG questionnaire was significant.

In line with the present study, Lemke et al.’s (2017) cross-sectional study on 262 long vehicle drivers showed that almost 60% of the subjects had MetS criteria, while driving experience and sleep quality was associated with the prevalence and severity of MetS [50]. Also, Huang et al. (2016) indicated that professional bus and taxi drivers had a higher prevalence of MetS than drivers of passenger cars [48]. In addition, Bowman et al. (2019) assessed sleep quality by Pittsburgh Sleep Quality Index. They indicated longer sleep latency related to MetS but the polysomnography studies were not significantly related to MetS [21].

The strength of our study is to examine a large population of professional drivers, hence our results are generalizable to general population of drivers. Moreover, professional driving is a common job in other cities of Iran.

### Limitations

This is a cross- sectional study, we needed laboratory test results beside Clinical and anthropometric measurements. Clinical evaluation of sleep through polysomnography is a more reliable way to estimate prevalence of OSA in professional drivers. In this study sleep disorder assessments were not clinically assessed. It is preferable if both, objective and subjective assessment of sleep and OSA assessment is done comprehensively. Fear of job loss or further police investigations may affect the self-reporting assessment (especially ESS questionnaire). Finally, it is worth nothing that our study lacks data from women population.

## Conclusion

Given the popularity of driving as a profession in Iran and high mortality and morbidity in crashes, drivers’ health is a major factor in reducing mortality rates. Sleepiness during driving is an important risk factor for traffic collisions, and the physical health of drivers affects it. MetS, characterized by abdominal obesity, hypertension and hypertriglyceridemia, causes sleep disorders in drivers. According to the results of this study, drivers with MetS suffer from more sleep disorders. A large number of publications has found conflicting results with that of ours stating that sleep disorders can influence MetS. We hypothesize that, in fact, there is a bi-directional association between sleep disorders and Mets. Thus, in this population, more attention should be paid to periodic occupational medical examination, as well as short-term and long-term training courses. Moreover, some controllable risk factors for sleep disorders and MetS, such as smoking, can be mitigated by acculturalization. As well, after identifying people with MetS, their families should be trained in the prevention and treatment of MetS. Researchers suggest that clinical trials should be conducted on sleep disorders associated with MetS to obtain more accurate results.

## Data Availability

The datasets used and/or analysed during the current study are available from the corresponding author on reasonable request.

## Footnotes

1 A questionnaire measuring snoring, tiredness, observed apnea, blood pressure, body mass index, age, neck circumference and gender; a full description is given below.

2 High Density Lipoprotein, Low Density Lipoprotein, Triglyceride, cholesterol, and Fasting Blood Sugar

3 **S**noring, **T**iredness, **O**bserved apnea, high blood **P**ressure, **B**MI, **A**ge, **N**eck circumference, **G**ender

## References

[1] Ford ESJD (2005). Risks for all-cause mortality, cardiovascular disease, and diabetes associated with the metabolic syndrome: a summary of the evidence. Diabetes Care.

[2] Gu D, Reynolds K, Wu X, Chen J, Duan X, Reynolds RF (2005). Prevalence of the metabolic syndrome and overweight among adults in China. Lancet.

[3] Zhu B, Haruyama Y, Muto T (2015). Yamazaki TJJoe. Association between eating speed and metabolic syndrome in a three-year population-based cohort study. J Epidemiol.

[4] Lopez A, Murray CC (1998). The Global Burden of Disease, 1990–2020. Nat Med.

[5] Grundy SM, Brewer HB, Cleeman JI, Smith SC, Lenfant CJC (2004). Definition of metabolic syndrome: report of the National Heart, Lung, and Blood Institute/American Heart Association conference on scientific issues related to definition. Circulation.

[6] Bansilal S, Castellano JM, Fuster VJI (2015). Global burden of CVD: focus on secondary prevention of cardiovascular disease. Int J Cardiol.

[7] Azizi F, Madjid M, Rahmani M, Emami H, Mirmiran P, Hadjipour RJI (2000). Tehran lipid and glucose study (TLGS): rationale and design. Iran J Endocrinol Metab.

[8] Shahbazian H, Latifi SM, Jalali MT, Shahbazian H, Amani R, Nikhoo A (2013). Metabolic syndrome and its correlated factors in an urban population in south west of Iran. J Diabetes Metab Disord.

[9] Kaykhaei M, Hashemi M, Narouie B, Shikhzadeh A, Jahantigh M, Shirzaei E (2012). Prevalence of metabolic syndrome in adult population from Zahedan, Southeast Iran. Iran J Public Health.

[10] Ebrahimi MH, Delvarianzadeh M, Saadat SJD (2016). Prevalence of metabolic syndrome among Iranian occupational drivers. Diabetes Metab Syndr.

[11] Korcarz CE, Stein JH, Peppard PE, Young TB, Barnet JH, Nieto FJJS (2014). Combined effects of sleep disordered breathing and metabolic syndrome on endothelial function: the Wisconsin sleep cohort study. Sleep.

[12] Cornier Marc-Andre, Dabelea Dana, Hernandez Teri L., Lindstrom Rachel C., Steig Amy J., Stob Nicole R., Van Pelt Rachael E., Wang Hong, Eckel Robert H. (2008). The Metabolic Syndrome. Endocrine Reviews.

[13] Peppard PE, Young T, Palta M, Skatrud JJNEJM (2000). Prospective study of the association between sleep-disordered breathing and hypertension. N Engl J Med.

[14] Nieto FJ, Herrington DM, Redline S, Benjamin EJ, Robbins JAJA, Medicine cc (2004). Sleep apnea and markers of vascular endothelial function in a large community sample of older adults. Am J Respir Crit Care Med.

[15] Coughlin SR, Mawdsley L, Mugarza JA, Calverley PM, Wilding JPJE (2004). Obstructive sleep apnoea is independently associated with an increased prevalence of metabolic syndrome. Eur Heart J.

[16] Tasali E (2008). Ip MSJPotATS. Obstructive sleep apnea and metabolic syndrome: alterations in glucose metabolism and inflammation. Proc Am Thorac Soc.

[17] Kasapis C, Thompson PDJJACC (2005). The effects of physical activity on serum C-reactive protein and inflammatory markers: a systematic review. J Am Coll Cardiol.

[18] Botoseneanu A, Chen H, Ambrosius WT, Allore HG, Anton S, Folta SC (2017). Effect of metabolic syndrome on the mobility benefit of a structured physical activity intervention—the lifestyle interventions and Independence for elders randomized clinical trial. J Am Geriatr Soc.

[19] Browne RAV, Farias-Junior LF, Freire YA, Schwade D, Macêdo GAD, Montenegro VB (2017). Sedentary occupation workers who meet the physical activity recommendations have a reduced risk for metabolic syndrome. J Occup Environ Med.

[20] Jahangiry L, Montazeri A, Najafi M, Yaseri M, Farhangi MJN (2017). An interactive web-based intervention on nutritional status, physical activity and health-related quality of life in patient with metabolic syndrome: a randomized-controlled trial (The Red Ruby Study). Nutr Diabetes.

[21] Bowman MA, Duggan KA, Brindle RC, Kline CE, Krafty RT, Thayer JF (2019). Prospective associations among objectively and subjectively assessed sleep and the metabolic syndrome. Sleep Med.

[22] Wang F, Xiong X, Xu H, Huang H, Shi Y, Li X, et al. The association between obstructive sleep apnea syndrome and metabolic syndrome: a confirmatory factor: analysis. Sleep Breath = Schlaf Atmung. 2019:1–9. 10.1007/s11325-019-01804-8 30820851

[23] Gaston SA, Park YM, McWhorter KL, Sandler DP, Jackson CL (2019). Multiple poor sleep characteristics and metabolic abnormalities consistent with metabolic syndrome among white, black, and Hispanic/Latina women: modification by menopausal status. Diabetol Metab Syndr.

[24] Lian Y, Yuan Q, Wang G, Tang F (2019). Association between sleep quality and metabolic syndrome: a systematic review and meta-analysis. Psychiatry Res.

[25] Catalán P, Martínez A, Herrejón A, Chiner E, Martínez-García MÁ, Sancho-Chust JN (2012). Internal consistency and validity of the Spanish version of the “Quebec sleep questionnaire” quality-of-life questionnaire for obstructive sleep apnea. Arch Bronconeumol (English Edition).

[26] Ebrahimi MH, Sadeghi M, Dehghani M, Niiat KSJIJOMEH (2015). Sleep habits and road traffic accident risk for Iranian occupational drivers. Int J Occup Med Environ Health.

[27] Monsef Kasmayi V, Assadi P, Maleki Ziabari SMJI (2014). The epidemiologic of the traffic accidents helped by EMS, Guilan 2011-2013. IJFM.

[28] Park JEJT (1970). Textbook of preventive and social medicine. (A treatise on community health.). Textbook of preventive and social medicine(A treatise on community health).

[29] Babamiri M, Javdan M, Dehghani M, Baryaji H, Abbasi MJJLS (2012). The study of the relationship between sensation seeking and type a personality with doing deliberate and unintentional violation in driving. J Life Sci Biomed.

[30] Gnardellis C, Tzamalouka G, Papadakaki M, Chliaoutakis JEJTF (2008). An investigation of the effect of sleepiness, drowsy driving, and lifestyle on vehicle crashes. Transport Res F: Traffic Psychol Behav.

[31] Peden M, Scurfield R, Sleet D, Mohan D, Hyder AA, Jarawan E (2004). World report on road traffic injury prevention.

[32] Ellen RL, Marshall SC, Palayew M, Molnar FJ, Wilson KG, Man-Son-Hing MJJ (2006). Systematic review of motor vehicle crash risk in persons with sleep apnea. J Clin Sleep Med.

[33] Philip P, Sagaspe P, Lagarde E, Leger D, Ohayon MM, Bioulac B (2010). Sleep disorders and accidental risk in a large group of regular registered highway drivers. Sleep Med.

[34] Talbott E, Helmkamp J, Mathews K, Kuller L, Cottington E, Redmond GJA (1985). Occupational noise exposure, noise-induced hearing loss, and the epidemiology of high blood pressure. Am J Epidemiol.

[35] Huang PLJD (2009). A comprehensive definition for metabolic syndrome. Dis Model Mech.

[36] Hwang JH, Kam S, Shin J-Y, Kim J-Y, Lee K-E, Kwon G-H (2013). Incidence of metabolic syndrome and relative importance of five components as a predictor of metabolic syndrome: 5-year follow-up study in Korea. J Korean Med Sci.

[37] Huang PL (2009). A comprehensive definition for metabolic syndrome. Dis Model Mech.

[38] Li Y, Yatsuya H, Iso H, Tamakoshi K, Toyoshima H (2010). Incidence of metabolic syndrome according to combinations of lifestyle factors among middle-aged Japanese male workers. Prev Med.

[39] Alberti KGMM, Zimmet P, Shaw J (2006). Metabolic syndrome—a new world-wide definition. A consensus statement from the international diabetes federation. Diabet Med.

[40] Haghighi KS, Montazeri A, Mehrizi AK, Aminian O, Golkhandan AR, Saraei M (2013). The Epworth sleepiness scale: translation and validation study of the Iranian version. Sleep Breath.

[41] Gay PCJR (2010). Sleep and sleep-disordered breathing in the hospitalized patient. Respir Care.

[42] Sadeghniiat-Haghighi K, Montazeri A, Khajeh-Mehrizi A, Ghajarzadeh M, Alemohammad ZB, Aminian O (2015). The STOP-BANG questionnaire: reliability and validity of the Persian version in sleep clinic population. Qual Life Res.

[43] Chung F, Abdullah HR, Liao PJC (2016). STOP-Bang questionnaire: a practical approach to screen for obstructive sleep apnea. Chest.

[44] Guglielmi O, Magnavita N, SJSp G (2018). Sleep quality, obstructive sleep apnea, and psychological distress in truck drivers: a cross-sectional study. Soc Psychiatry Psychiatr Epidemiol.

[45] Anund A, Ihlström J, Fors C, Kecklund G, Filtness AJI (2016). Factors associated with self-reported driver sleepiness and incidents in city bus drivers. Ind Health.

[46] Gu G, Yu S, Zhou W, Wu H, Kang L, Chen RJZZC (2017). Sleep quality and occupational stress relationship analysis of 1413 train drivers in a railway bureau. Chin J Ind Hyg Occup Dis.

[47] Soleimanloo SS, White MJ, Garcia-Hansen V, Smith SSJP (2017). The effects of sleep loss on young drivers’ performance: A systematic review. PLoS One.

[48] Huang H, Wang W, Zhou J, Li Q, Feng W, Wu ZJZZC (2016). Metabolic syndrome and its influencing factors in professional automobile drivers in a company. Chin J Ind Hyg Occup Dis.

[49] Mohebbi I, Saadat S, Aghassi M, Shekari M, Matinkhah M, Sehat SJP (2012). Prevalence of metabolic syndrome in Iranian professional drivers: results from a population based study of 12,138 men. PLoS One.

[50] Lemke M, Apostolopoulos Y, Hege A, Wideman L, Sönmez SJOM (2017). Work organization, sleep and metabolic syndrome among long-haul truck drivers. Occup Med.

